# Vitamin D levels, brain volume, and genetic architecture in patients with psychosis

**DOI:** 10.1371/journal.pone.0200250

**Published:** 2018-08-24

**Authors:** Akiah Ottesen Berg, Kjetil N. Jørgensen, Mari Nerhus, Lavinia Athanasiu, Alice B. Popejoy, Francesco Bettella, Linn Christin Bonaventure Norbom, Tiril P. Gurholt, Sandra R. Dahl, Ole A. Andreassen, Srdjan Djurovic, Ingrid Agartz, Ingrid Melle

**Affiliations:** 1 Norwegian Centre for Mental Disorders Research (NORMENT), K. G. Jebsen Centre for Psychosis Research, Institute of Clinical Medicine, University of Oslo, Oslo, Norway; 2 Division of Mental Health and Addiction, Oslo University Hospital, Oslo, Norway; 3 Department of Psychiatric Research, Diakonhjemmet Hospital, Oslo, Norway; 4 Akershus University Hospital, Division for Mental Health, Lørenskog, Norway; 5 Institute for Public Health Genetics, University of Washington, Seattle, Washington, United States of America; 6 Hormone Laboratory, Department of Medical Biochemistry, Oslo University Hospital, Oslo, Norway; 7 Department of Medical Genetics, Oslo University Hospital, Oslo, Norway; 8 NORMENT, KG Jebsen Centre for Psychosis Research, Department of Clinical Science, University of Bergen, Bergen, Norway; 9 Centre for Psychiatric Research, Department of Clinical Neuroscience, Karolinska Institutet, Stockholm, Sweden; University of Queensland, AUSTRALIA

## Abstract

**Background:**

Lower vitamin D levels are found in people with schizophrenia and depressive disorders, and also associated with neuroimaging abnormalities such as reduced brain volume in both animals and humans. Reduced whole brain and increased ventricular volume are also systematically reported in schizophrenia. Even though vitamin D deficiency has been proposed as a risk mechanism for schizophrenia there exist no studies to date of the association between vitamin D levels and brain volume in this population. Therefore, we investigated the relationship between vitamin D levels and brain phenotypes in psychotic disorders, and assessed possible interactions with genetic variants in vitamin D receptor (VDR) and other genetic variants that play a role in vitamin D levels in the body.

**Methods:**

Our sample consisted of 83 psychosis patients and 101 healthy controls. We measured vitamin D levels as serum 25-hydroxyvitamin D. All participants were genotyped and neuroimaging conducted by structural magnetic resonance imaging.

**Results:**

Vitamin D levels were significantly positively associated with peripheral grey matter volume in patients (β 860.6; 95% confidence interval (CI) 333.4–1466, p < .003). A significant interaction effect of *BSML* marker (*rs1544410*) was observed to mediate the association between patient status and both white matter volume (β 23603.3; 95% CI 2732.8–48708.6, p < .05) and whole brain volume (β 46670.6, 95% CI 8817.8–93888.3, p < .04). Vitamin D did not predict ventricular volume, which rather was associated with patient status (β 4423.3, 95% CI 1583.2–7267.8p < .002) and *CYP24A1* marker (*rs6013897*) (β 2491.5, 95% CI 269.7–4978.5, p < .04).

**Conclusions:**

This is the first study of the association between vitamin D levels and brain volume in patients with psychotic disorders that takes into account possible interaction with genetic polymorphisms. The present findings warrant replication in independent samples.

## Introduction

In the last decade, we have seen a growing interest in the possible interaction between vitamin D deficiency and the development or clinical expression of psychiatric disorders, in particular depression and schizophrenia. A number of environmental risk factors for schizophrenia and psychosis such as urban birth, winter birth, malnutrition, and migration from the southern to the northern hemisphere [1] are also associated with the risk of vitamin D deficiency, supporting this as an underlying risk factor for schizophrenia [2, 3]. To further investigate the role of vitamin D in the etiology of psychosis, this study aims to demonstrate whether serum 25-hydroxyvitamin D (S-25(OH)D) levels and genetic variants that interact with vitamin D levels are related to altered global brain volumes previously implicated in psychotic disorders.

Adults with schizophrenia and depression have been found to have lower serum 25-hyroxyvitamin D (S-25(OH)D) levels compared to healthy controls [4–6], which is the most stable measure of vitamin D from both dietary and environmental sources [7]. Meta-analyses have shown lower vitamin D levels in adults with schizophrenia [8] compared to healthy controls, however, some of the included studies did not control for potential confounding factors, such as ethnic minority status, which may have had an impact on the results and interpretation of these analyses [9]. Although there is some support for an association between vitamin D levels and diagnosis of schizophrenia, the causal pathways are complex and have yet to be described in full in the literature.

Findings from animal models suggest that vitamin D may serve important functions during neurodevelopment through supporting cell differentiation and inhibiting apoptosis [10]. Low levels of S-25(OH)D have been associated with anthropometric measures [11], and vitamin D depletion has been associated with smaller brain volume and larger ventricles in cross-sectional studies of both animals and humans [12]. Studies in the elderly have shown a positive association between vitamin D levels and whole brain volume [13], and a negative association with white matter abnormalities [14]. Intracranial volume, grey and cerebral white matter volume has also been associated with S-25(OH)D levels in young women [15]. Larger ventricles and reduced total brain volume are among the most consistently reported structural imaging factors found to be associated with schizophrenia in adults [16–19]. Also, a longitudinal study found higher S-25(OH)D3 levels to be associated with less regional cortical thinning in healthy participants within the age range of 23–87, over a three-year period [20], suggesting a protective effect of vitamin D. However, to date, no MRI study has examined the relationship between vitamin D levels and global brain volumes in psychotic disorders.

As a variety of brain phenotypes appear to be associated with vitamin D levels it is of potential importance to examine if these findings are mediated by genetic variation. A number of polymorphisms in genetic variants play a role in S-25(OH)D levels and are associated with vitamin D receptors (VDR), which are proteins expressed widely in the brain [21]. The *VDR* gene (ENSG00000111424) contains several polymorphisms that have been studied in relation to diseases such as osteoarthritis, diabetes, and cardiovascular disease (CVD), but far less information is available about its relation to brain structure and neuropsychiatric disorders [21]. We hypothesize that vitamin D plays a role in psychiatric disease pathways and brain structure variability through interaction with genetic variants associated with vitamin D in the literature [22–26].

In summary, low S-25(OH)D levels are frequently observed in patients with psychotic disorders and in groups at high risk of developing schizophrenia [27, 28], and have also been associated with decreased brain volume and larger ventricles in rodents and human adults [12]. However, whether this effect is more pronounced in patients with psychotic disorders is unknown, and the relationship between vitamin D deficiency, brain phenotypes, and psychiatric disease has yet to be determined. This study was designed to achieve the following aims: 1) to test the hypothesis that S-25(OH)D levels are positively correlated with total brain volume (both white and grey matter), and negatively correlated with ventricular size in patients with psychosis compared to healthy controls; and 2) to determine whether S-25(OH)D levels interact with genetic variants, to contribute to brain volume variation in patients with psychotic disorders compared to healthy controls.

## Materials and methods

The current study was conducted in Oslo, Norway (59°N) as part of the ongoing “Thematically Organized Psychosis” (TOP) Study at the Norwegian Centre for Mental Disorder Research (NORMENT). It was approved by the Regional Committee for Medical Research Ethics and the Norwegian Data Inspectorate. Our research methodology conformed to The Code of Ethics of the World Medical Association, Helsinki Declaration [29]. The study had a cross-sectional design including a large, non-selected consecutively recruited catchment area sample of patients with a DSM-IV schizophrenia or bipolar spectrum disorder (psychotic disorders) and a healthy control group. All participants gave written informed consent.

### Participants

The sample consisted of 83 patients and 101 healthy controls recruited between 2011 and 2014 that had S-25(OH)D measured, were genotyped, and had undergone magnetic resonance imaging (MRI). All participants had blood samples and MRI scans conducted within the same season or within 21 days of each other, of importance as S-25(OH)D serum levels vary between seasons and years. Of the 83patients 46 (55.4%) had a schizophrenia spectrum disorder, 36 (43.4%) had a bipolar spectrum disorder and 1 (1.2%) had another psychotic disorder. Inclusion criteria were age 18–65 years, IQ>70, no signs of organic etiology, substance-induced symptoms or severe trauma to the CNS, and the ability to understand and speak a Scandinavian language. To avoid confounding of the association between S-25(OH)D levels and anthropomorphic characteristics or other traits under investigation by population-associated genetic and environmental factors, we excluded participants with non-European ancestry from the current study.

Healthy control participants were randomly selected from national statistical records from the same catchment area and contacted by letter inviting them to participate. Inclusion criteria were no current or previous psychiatric disorders, no family history of severe psychiatric disorders, no alcohol or substance dependence, no head trauma and no use of cannabis in the 6 months preceding assessment.

### Assessments

Diagnoses in the patient group were assessed with the structured clinical interview for DSM-IV disorders (SCID-I) [30] by a trained psychologist or physician. All patients included were subject to a general somatic examination including body mass index (BMI) and fasting morning blood samples. Current medication at the time of MR scan was estimated by conversion into chlorpromazine (CPZ) equivalents [31], or if conversion values were not available two alternative sources were used [32, 33].

### Biochemical

From September 2012, total S-25(OH)D was determined using a liquid chromatography-tandem mass spectrometry (LC-MS/MS) method developed at the Hormone Laboratory (Oslo University Hospital, Aker, Norway) [34]. Prior to September 2012, total S-25(OH)D was measured by radioimmunoassay (RIA [kit from Diasorin]) [35] in the same laboratory. The regression equation LC-MS/MS = 1.16 × (RIA) − 9 was obtained at the laboratory during method comparison and was used to convert all S-25(OH)D concentrations obtained by LC-MS to equivalent concentrations obtained by RIA, which were used in the analyses.

### MRI acquisition

MRI images were obtained at Ullevål, Oslo University Hospital, using a General Electric Signa HDxt 3T system equipped with an 8-channel head coil. 3D sagittal fast spoiled gradient echo (FSPGR) volumes were obtained using the FSPGR_SAG_TI450 sequence (TE: Min full, TR: 7.8s, TI: 450 ms, flip angle: 12°; FOV = 25.6 cm, voxel size = 1.0x1.0x1.2 mm^3^). No acceleration by parallel imaging was applied. Patients and controls were scanned interchangeably to avoid any potential bias related to scanner drift.

### MRI processing: SIENAX

Normalized whole brain volume and global gray and white matter volumes (*normalized* whole brain, white matter, grey matter, peripheral grey matter volume and total ventricular volume) were calculated using SIENAX [36], which is part of the FSL software package [37], v. 5.0. SIENAX extracts brain and skull images from the single whole head input data. For this study, the brain extraction parameters were first varied systematically in 10 independent images, since optimal parameters may depend on scanner and sequence. We found a fractional intensity threshold of 0.35 to be optimal. Bias field filtering and neck removal was applied. The brain image was then affine-registered to MNI152 space using the skull image to determine the registration scaling in order to obtain the volumetric scaling factor, which is used to normalization for head size. Next, tissue-type segmentation with partial volume estimation (FAST) [38] was carried out in order to calculate total volume of brain tissue. Using this method, measures of global brain volumes both before and after normalization for head size were obtained. SIENAX is fully automated and no manual editing was performed, however, all volumes were visually inspected to ensure quality.

### Genotyping and quality control

Genomic DNA from participants was extracted from peripheral whole blood, and genotyped at deCODE Genetics (Reykjavik, Iceland) on either the Human OmniExpress-12v-1-1_B (Illumina, San Diego, CA, USA) or Human OmniExpress-12v1_H (Illumina, San Diego, CA, USA) platforms in accordance with the manufacturer’s standard protocol. Genotypes were assigned in concordance with the standard Illumina protocol in GenomeStudio software V2011.1 version 1.9.4. We performed a standard pre-imputation quality control, where markers exhibiting high rates of genotyping missingness (>95%), minor allele frequency (MAF) <1% or showing departure from the Hardy Weinberg equilibrium (p<1.00E-05) were excluded from the analyses. Also, individuals exhibiting high rates of genotyping missingness (>5%), cryptic relatedness (PI_HAT>15%) or genome-wide heterozygosity (outside mean ± 4SD of the sample) were removed from the analyses, as well as individuals with incoherent sex assessment based on the homozygosity estimate of X chromosome markers implemented in PLINK [39]. Finally, we restricted our data by excluding individuals with non-European ancestry (outside 3SD range of either one of the first two genetic principal components).MACH (http://www.sph.umich.edu/csg/abecasis/MACH) was used to impute the genotypes onto the reference haplotypes from the 1000 Genomes Project (build 37, assembly Hg19).

#### Genetic variants of interest

Nine single-nucleotide polymorphism (SNP’s) markers of VDR previously associated with vitamin D levels; namely *rs2282679*, *rs7041*, *rs1790349*, *rs6599638*, *rs2060793*, *rs1544410*, *rs12785878*, *rs10741657*, *rs6013897* [22–26] were extracted from the imputed dataset (See Table 1).

**Table 1:** Description of single-nucleotide polymorphism (SNP) markers.

SNP ID	Chr. Position	Functional Region	Genomic Region	Genome Build	Gene ID	Study
rs2282679	chr4:72608383	intronic	4q13.3	hg19/Human	GC	*Ahn*, *2010*
rs7041	chr4:72618334	coding	4q13.3	hg19/Human	GC	*Ahn*, *2010*
rs1790349	chr11:71142350	intergenic	11q13.4	hg19/Human	none	*Ahn*, *2010*
rs6599638	chr10:124704149	intronic	10q26.13	hg19/Human	C10orf88	*Ahn*, *2010*
rs2060793	chr11:14915310	intergenic/upstream	11p15.2	hg19/Human	upstream CYP2R1	*Ahn*, *2010*
rs1544410	chr12:48239835	intronic	12q13.11	hg19/Human	VDR	*Lambrinoudaki*, *2013*
rs12785878	chr11:71167449	intronic	11q13.4	hg19/Human	NADSYN1	*Wang*, *2010*
rs10741657	chr11:14914878	intergenic/upstream	11p15.2	hg19/Human	upstream CYP2R1	*Wang*, *2010*
rs6013897	chr20:52742479	intergenic	20q13.2	hg19/Human	implicated in locus for CYP24A1	*Wang*, *2010*

### Statistical analyses

The statistical analyses were performed in SPSS 22 [40]. The level of significance was pre-set to <0.05, two sided since our research questions were strictly hypothesis-based. We removed two outliers from the healthy control group with high S-25(OH)D levels. T-tests and chi-square tests were used to compare patients and controls on socio-demographic measures of age, ethnic minority status, gender, height, weight, BMI, baseline S-25(OH)D levels and normalized brain and ventricular volumes. For the statistical analyses of genetic variants implicated in the vitamin D pathway, we created an additive variable (0,1,2) for each respective number of minor/risk alleles (heterozygote: 1, homozygote: 2). In the whole sample Pearson’s partial correlation test was conducted between all three variables of interest; vitamin D levels, normalized brain volume and SNPs. In patients we conducted Pearson’s correlations between normalized brain levels with dosage of antipsychotic medication (CPZ).

Prediction models were tested with multiple linear regressions. We analyzed the relationship between S-25(OH)D levels and each SNP of interest on relevant normalized brain volume measures while controlling for age, male gender, height/weight or BMI, patient/control and genetic principal components, removing the variables that did not contribute to the model significantly. The regression models only included SNP’s that were found to be significantly associated with brain volume in the preliminary tests. Height has previously been linked to the VDR SNP *rs1544410* [41, 42] and was added to models including this marker instead of BMI. We also assessed possible interaction effects in this order: S-25(OH)D-by-VDR marker, patient-by-S-25(OH)D and patient-by-VDR marker. The regression analyses were re-estimated using bootstrapping procedure with iteration of 1000 (CI95%) to control for multiple testing.

## Results

Table 2 contains a description of the final sample in terms of gender, age, height, weight, BMI, S-25(OH)D levels and normalized brain and ventricular volumes, as well as comparative analyses between patients and healthy controls. Patients had statistically significant higher BMI and larger ventricles than the healthy control group. S-25(OH)D levels did not differ between groups.

**Table 2:** Sample description and patient/healthy control comparison.

Variable	Complete sample (N = 184)	Patients (N = 83)	Controls (N = 101)	x2	p>
Male	103 (56%)	44 (53%)	59 (57%)	.540	ns
Chlorpromazine-equivalent dose					
Current (N = 56)		287.9 ± 191.1			
First generation (N = 5)		56.1 ± 32.4			
Second generation (N = 55)		288.02 ± 190.13			
				**t-test**	**p>**
Age	30.53 ± 9.42	29.4 ± 10.9	31.5 ± 7.9	1.487	ns
Height (cm)	176.3 ± 9.8	176 ± 9.7	176.5 ± 9.9	.306	ns
Weight (kg)	78.3 ± 16.6	80 ± 19	76.8 ± 14.3	-1.265	ns
BMI	24.4 ± 6.0	25.7 ± 5.3	23.3 ± 6.3	2.713	.007
S-25(OH)D nmol/L	53.03 ± 19.3	51.9 ± 21.3	53.99 + 17.5	.743	ns
Grey matter volume	653801.3 ± 58269.3	649285.5 ± 64822.7	657512.4 ± 52315.9|	.953	ns
White matter volume	561997.0 ± 60617.2	556135.3 ± 61447.7	566813.9 ± 59800.7	1.190	ns
Whole brain volume	1215798.2 ± 110268.1	1205420.8 ± 115583.1	1224326.3 ± 105515.2	1.158	ns
Peripheral grey volume	514597.4 ± 47308.1	510882.6 ± 52809.9	517650.2 ± 42283.3	.965	ns
Total ventricular volume	26060.4 ± 9768.5	28133.9 ± 10894.6	24356.5 ± 8413.9	2.653 -	.009

Table 3 shows correlations between S-25(OH)D and normalized whole brain, white matter, grey matter, peripheral grey matter volume and total ventricular volume before adjustment for age, gender and height. There were no significant associations for the analyses of the whole sample. Stratifying for patient versus control revealed that S-25(OH)D levels correlated positively with white matter (p < .02), whole brain (p < .02) and peripheral grey matter (p < .05) volumes in the patient group but not in the controls.

**Table 3:** Pearson’s correlations for S-25(OH)D and brain volume stratified for patients and healthy controls.

Brain volume	Total (N = 184)	Patients (N = 83)	Controls (N = 101)
Grey matter volume	0.083	0.212	-0.083
White matter volume	0.135	.261*	-0.002
Whole brain volume	0.118	.257*	-0.042
Peripheral grey volume	0.095	.234*	-0.087
Total ventricular volume	0.052	0.011	0.133

*. Correlation is significant at the 0.05 level (2-tailed).

We found no significant associations between level of medication use (measured as CPZ equivalents) and neither S-25(OH)D levels nor measures of brain volume, the exception being a negative correlation between CPZ for first generation anti-psychotics and grey matter volume (r = -.885, p < .05) (data not shown). This measure was, however, based on the only 5 participants currently on first generation anti-psychotic medication, and therefore not controlled for in further analyses.

The results for associations between S-25(OH)D levels and genotypes can be found in Table 4. S-25(OH)D levels were significantly associated with SNPs *rs2282679* and *rs7041*. Positive correlations were found between the *rs1544410* marker with white matter (r = .206, p < .01) and whole brain volume (r = .159, p < .05), and the *rs6013897* marker and total ventricular volume (r = .202, p < .01). Based on these analyses, we selected and analyzed *rs1544410* and *rs6013897* as candidates for variants that may influence the association between vitamin D and brain volume phenotypes.

**Table 4:** Pearson’s correlation between genotype and S-25(OH)D nmol/L.

SNP	Genotype	S-25(OH)D (nmol/L)
rs2282679 (N = 181)	GG (N = 17)	-.211*
	GT (N = 78)	
	TT (N = 86)	
rs7041	AA (N = 43)	-.214*
	AC (N = 88)	
	CC (N = 53)	
rs1790349 (N = 183)	CC (N = 6)	-.050
	CT (N = 56)	
	TT (N = 121)	
rs6599638	AA (N = 50)	.126
	AG (N = 85)	
	GG (N = 49)	
rs2060793	AA (N = 26)	.069
	AG (N = 88)	
	GG (N = 70)	
rs1544410 (N = 182)	TT (N = 29)	.015
	CT (N = 94)	
	CC (N = 59)	
rs12785878	TT (N = 87)	.048
	GT (N = 81)	
	GG (N = 16)	
rs10741657	AA (N = 26)	.083
	AG (N = 90)	
	GG (N = 68)	
rs6013897 (N = 182)	AA (N = 7)	-.089
	AT (N = 74)	
	TT (N = 101)	

* p < .05

The results of the regression analyses are shown in Table 5. White matter volume was predicted by gender, height and interaction between patient status and rs1544410. Similar results were found for whole brain volume with age, gender, height and interaction between patient status and rs1544410 as significant predictors. Peripheral grey matter was predicted by a significant interaction between patient status and S-25(OH)D levels, with a positive linear correlation in patients but an inverse relationship in controls. Total ventricular volume was predicted by age, gender, patient status and *rs6013897* but not current S-25(OH)D levels, and we found no significant interaction effects.

**Table 5:** Multiple linear regression models predicting white—and peripheral grey matter, whole brain—and total ventricular volume.

Model	B	Bias	Std.error	p	Lower Bound	Upper Bound	AdjR^2^	F	p
**White matter volume**							.415	16.450	.001
(Constant)	340838.18	-3494.79	105039.76	0.003	130649.24	542924.85			
Age	25.24	33.16	376.85	0.954	-639.18	830.44			
Gender	-48151.73	106.04	10136.10	0.001	-68847.43	-28135.19			
Height	1476.89	7.39	527.27	0.005	420.73	2512.84			
Weight	-60.86	7.17	224.13	0.780	-489.44	394.42			
Patients/Healthy controls	-50082.94	-843.74	20090.17	0.011	-91352.95	-13026.73			
S-25(OH)D nmol/L	253.42	-1.46	165.63	0.121	-102.22	562.21			
rs1544410	-26010.80	-960.36	17313.97	0.133	-61650.81	5686.39			
Patient X rs1544410	23603.27	705.00	11438.90	0.049	2732.79	48708.57			
**Whole brain volume**							.435	17.776	.001
(Constant)	775332.29	-9265.97	191297.38	0.001	378907.95	1146070.28			
Age	-2603.32	31.94	701.37	0.001	-3917.78	-1139.46			
Gender	-84566.40	1053.37	18777.35	0.001	-122618.72	-46054.27			
Height	3606.92	22.17	978.00	0.001	1751.69	5619.78			
Weight	-655.83	31.48	451.70	0.138	-1459.25	334.49			
Patients/Healthy controls	-101398.72	-2074.77	37603.33	0.006	-179341.57	-34555.99			
S-25(OH)D nmol/L	454.78	-7.58	316.26	0.151	-183.69	1047.28			
rs1544410	-58598.44	-2160.59	32413.00	0.069	-127301.79	306.74			
Patient X rs1544410	46670.61	1427.79	21458.00	0.036	8817.77	93888.34			
**Total ventricular volume**							.207	8.898	.001
(Constant)	21582.07	69.10	5194.50	0.001	11406.62	31759.04			
Age	232.46	-8.72	98.70	0.018	46.08	419.04			
Gender	-5893.24	71.08	1182.73	0.001	-8252.21	-3502.80			
BMI	32.78	5.12	130.71	0.807	-219.46	305.41			
Patients/Healthy controls	4423.31	-43.91	1443.68	0.002	1583.22	7267.76			
S-25(OH)D nmol/L	34.39	-0.63	34.64	0.308	-34.92	100.93			
rs6013897	2491.48	7.22	1154.52	0.032	269.73	4978.46			
**Peripheral grey matter volume**							.430	23.980	.001
(Constant)	694849.08	-1278.81	19810.55	0.001	655363.06	733425.36			
Age	-2295.30	19.45	278.56	0.001	-2844.28	-1741.52			
Gender	-46235.64	288.21	5370.17	0.001	-56573.78	-35150.20			
BMI	-1271.56	24.57	378.32	0.001	-2052.17	-557.05			
Patients/Healthy controls	-51039.94	-180.59	14932.30	0.001	-82662.66	-22629.55			
S-25(OH)D nmol/L	-1043.07	-11.12	444.49	0.017	-1982.31	-226.77			
Patient X S-25(OH)D nmol/L	860.56	6.76	290.93	0.003	333.42	1465.95			

Results are presented after bootstrapping procedure with iteration of 1000 (CI95)

## Discussion

This study aimed to elucidate the association between S-25(OH)D levels and brain volume in psychotic patients compared to healthy controls, and investigated possible interactions with genetic polymorphisms. Whole brain and white matter volumes were significantly associated with an interaction between the *VDR BSML* marker *rs1544410* and patient status, but not with current S-25(OH)D levels. Ventricular size was predicted by the *CYP24A1* SNP, *rs6013897*, but not current S-25(OH)D levels. Peripheral grey matter volume was the only brain phenotype in this study to be associated with S-25(OH)D levels, but only in patients. Our results provide novel insight into the relationship between brain phenotypes, genomic variants, and S-25(OH)D levels, as studies in this field have to date been few and with conflicting results.

### Neuroimaging and genetic determinants of vitamin D

Previous research has found a significant association between S-25(OH)D levels and brain volumes [12]. Reduced brain volume in those with vitamin D deficiency is shown in one animal study [43] and in a cross-sectional human study of community dwellers older than 65 [44], but not in other studies of animals [45] or elderly humans [46]. Except for in concern to peripheral grey matter this was not replicated in our study. We found that the apparent association of current S-25(OH)D levels with white-and whole brain matter did not survive correction for confounders such as age, gender, height and patient status. We did however find an interaction between patient status and genetic variants, for white matter and whole brain volume (*rs1544410*) and ventricular volume (*rs6013897*).

The *rs1544410* is found to influence individual responses to vitamin D supplementation [25, 26], and has been studied extensively in association with a number of human diseases, among others endocrine immune-mediated disorders [47]. This is of interest because of the associations found between the immune system and risk of schizophrenia [48]. Even though this marker is not previously linked to brain morphology it could have a mediating role that deserves further attention. No association is found between *rs1544410* markers and heightened risk of psychosis in case control studies [24], however it is associated with cognitive decline among healthy women [49]. As cognitive deficits are a hallmark symptom of psychotic disorders this is a marker of interest.

### Vitamin D and ventricular volume

Contrary to previous research, we did not find an association between S-25(OH)D levels and total ventricular volume. A systematic review and meta-analyses concluded that vitamin D depletion is associated with greater lateral ventricular volume [50]. However, these findings were based on animal studies of vitamin D deficiency during gestation, hence affecting the developing brain [43, 45], and cross-sectional studies of humans over the age of 60 or with dementia [51, 52]. One study found that healthy elderly Caucasians with vitamin D deficiency (S-25(OH)D≤50 nmol/L) had 28% larger lateral ventricles than those with sufficient S-25(OH)D levels [51]. The elderly have a particularly high risk for vitamin D deficiency [53] and aging is associated with enlargement of the lateral ventricles [54]. The mean age in our sample, however, was only 30 years. A more age similar sample of healthy young women (mean age 22), with relatively high S-25(OH)D levels, did not find an association with ventricular volume, rendering age as a possible explanation for the differences found between studies [15]. Therefore, further population studies in different age ranges are needed before we can conclude that there exists an age-independent correlation between S-25(OH)D levels and ventricular size.

Our findings could suggest that factors other than current vitamin D levels influence total ventricular volume in patients with psychosis, as we found this measure to be associated with *CYP24A1* polymorphisms (*rs6013897)*. This has not been reported nor assessed in previous studies of S-25(OH) D levels and brain volume, and should be the subject of future research. *CYP24A1* is a candidate gene for S-25(OH)D concentration and among the genetic variants near genes involved in cholesterol synthesis, hydroxylation and vitamin D transport that affect vitamin D status [23]. This gene encodes a member of the cytochrome P450 superfamily of enzymes which catalyze many reactions involved in drug metabolism as well [55], and could thus be of interest in psychopharmacological studies of patients with psychosis.

Further, baseline DNA methylation levels of *CYP24A1* predict variation in vitamin D response in previous studies [56]. We found that polymorphisms in *CYP24A1* and not current vitamin D levels, were associated with ventricular volume and suggest that a genetic contribution to response to vitamin D levels during early development may be associated with ventricular volume. This process would thus affect the developing brain but not current vitamin D status as such in an adult population. Another possibility is that the *CYP24A1* SNP has a subtle impact on vitamin D levels over the lifespan, such that repeated measurements would be needed to detect the between-subject variation in S25-OH D levels caused by variation in this SNP.

### Vitamin D and neurodevelopment

Animal models have shown that developmental vitamin D deficiency may cause abnormal brain development contributing to sensitivity to agents that induce psychosis [57] and adverse neuropsychiatric outcome [58]. It has also been found that neonatal S-25(OH)D3 levels are associated with increased risk of schizophrenia [59], and that vitamin D supplements during the first year of life decreased the risk of schizophrenia in male offspring [60].

An individual’s susceptibility to disease in adulthood is linked to prenatal phases of development, particularly through placental function [61]. Flow of nutrients such as vitamin D in the placenta programs physiological systems at the genetic, cellular, tissue, organ and system level, thus influencing structural and functional development in the fetus [62], and placental insufficiency is associated for example with enlarged ventricles and reduced brain weight in animal models [63]. Vitamin D metabolism in the placenta also modulates cytokine production and the immune response to infection, as well as the balance between adaptive and innate maternal immune systems [64].

Vitamin D deficiency in utero is thus associated with brain development, the immune system and increased risk of schizophrenia, commonly regarded as a neurodevelopmental disorder, even though the symptoms of schizophrenia usually do not manifest until puberty. Vulnerability in the immune system may lay latent until major changes in the endocrine and immune system occur during puberty [65] and when an individual is exposed to environmental stressors [66]. Animal models show that vitamin D deficiency exacerbates vulnerability to social stress [67] and that prenatal insult, with the ensuing immune activation, increases vulnerability to stress [68].

The findings of patient-by-genetic marker interaction on brain volume found here could suggest that S-25(OH)D levels resulting from genetic expression during neural development influence brain volume and perhaps continue to influence S-25(OH)D concentrations later in life. Our results support the hypothesis that the association between vitamin D and brain morphology in psychosis manifests during early development through genetic mechanisms interacting with the environment as part of a multifactorial etiology of schizophrenia. Future studies should assess the genetic influence of polymorphisms in *BSML* and *CYP24A1* markers on vitamin D levels in utero, and how this effects the development of the immune system and later vulnerability to environmental stress.

### Current vitamin D levels and grey matter

We did find that current S-25(OH)D levels were associated with peripheral grey matter volume. Dysmaturation of grey matter has been suggested as a partial explanation of early and late neural developmental abnormalities common in psychosis [69]. A steeper rate of cortical grey matter decline is consistently found in persons who convert from clinical high risk of psychosis to frank psychosis and thought to be associated with neuroinflammation [70]. Also, it has been demonstrated that vitamin D has a neuroprotective effect in animal models of Parkinson’s disease by attenuating pro-inflammatory and up-regulating anti-inflammatory processes [71]. In research on multiple sclerosis (MS) an association was found between vitamin D levels and inflammatory activity, and more recently a possible neuroprotective role of vitamin D on grey matter volume [72]. Our findings lend support to the hypothesis that vitamin D has a neuroprotective effect on grey matter. Candidate genes for schizophrenia are related to immune function [73] and findings of the interaction between inflammation, vitamin D and brain morphology suggest a complex interplay of genes and environment in neurodevelopment. At the moment it is uncertain if vitamin D levels may have an etiological influence on psychosis, MS or Parkinson’s disease, but research supports a possible prognostic effect on MS in terms of severity and relapse rates [74]. Vitamin D supplements as an add-on therapy in psychosis are deemed warranted and we suggest a possible effect on grey matter volume, inflammation and cognitive and negative symptoms of the disorder [75, 76].

This study also found two genetic variants to be significantly associated with current S-25(OH)D levels in patients and controls, *rs2282679* and *rs7041* located in the *GC* gene on chromosome 4. While it is possible that each SNP independently has an effect on S-25(OH)D levels, the most parsimonious explanation is that one SNP is driving the association while the other demonstrates an association with vitamin D because of its high Linkage Disequilibrium (LD) with the true causal SNP. A third explanation is that neither SNP is causally associated with S-25(OH)D levels, but both are in LD with a third (unobserved) variant, which is the true causal one. Neither of these markers was associated with brain volume and therefore of any further interest to this study, but both could be of interest to vitamin D research in general.

### Limitations

The findings of the present study should be interpreted in light of some limitations. First, the study is cross-sectional which makes it difficult to draw conclusions about causal relationships. We had strict inclusion criteria selecting only participants who had blood samples and MRI conducted within the same season or within 21 days. However, there is no established standard in the field regarding how quickly S-25(OH)D levels change over time. Other studies of S-25(OH)D levels and MRI have had varying time intervals between blood sampling and imaging; from a few hours [51] up to 30 days in between [77, 78]. Our strict criteria resulted in a small sample size and findings should be replicated in larger, independent samples. We controlled for a number of factors of importance such as ancestry, gender, age, height, weight, BMI and patient status but other potential confounders may exist. Also, our study focused on global brain measures, and we cannot exclude associations between S-25(OH)D levels and more regional brain structures. In schizophrenia S-25(OH)D levels were found to be associated with hippocampal grey matter volume, but not with other regional volumes [79], and there may be an age related decline in the association between S-25(OH)D levels and total hippocampal volume in patients with psychotic disorder [80]. Longitudinal studies throughout the lifespan are needed to assess the role of vitamin D on brain morphology.

Lower S-25(OH)D levels have previously been found in patients with psychosis but some studies suggest that this is driven by ethnic minority status and not patient status [34]. In this study, we removed participants with non-European ancestry as external factors that are associated with ancestry and S-25(OH)D levels could be a confounder, and because of the reduced accuracy of imputation of these samples in individuals of non-European ancestry descent. Genetic models of common diseases or traits derived from EA populations may generate spurious results in non-European ancestry populations due to differences in environmental factors, allele frequencies and varying effect sizes between populations [81]. Our exclusion of non-European populations is common in genomics research, but a significant weakness that must be addressed in future studies by greater inclusion of diverse ancestral groups, in an effort to provide equal access to genomic medicine and ameliorate existing health disparities [82].

### Conclusions

To conclude, this study adds to the growing knowledge base of associations between vitamin D and brain morphology. Our first hypothesis was partially confirmed as S-25(OH)D levels were found to be positively associated with peripheral grey matter volume in patients. However, we did not find an association between S-25(OH)D levels and total ventricular volume as expected. The *CYP24A1* (*rs6013897*) locus was found to interact with patient status in predicting total ventricular volume. Further, the *VDR* locus *BSML* (*rs1544410*) interacted with patient status in predicting white matter and whole brain volumes. We encourage others to conduct similar and replication studies of our findings on vitamin D, genetic variants, and brain phenotypes.

## Supporting information

S1 Dataset Minimal anonymized dataset. Including legend.
(XLSX)
